# Hate speech detection: Challenges and solutions

**DOI:** 10.1371/journal.pone.0221152

**Published:** 2019-08-20

**Authors:** Sean MacAvaney, Hao-Ren Yao, Eugene Yang, Katina Russell, Nazli Goharian, Ophir Frieder

**Affiliations:** Information Retrieval Laboratory, Georgetown University, Washington, DC, United States of America; Tsinghua University, CHINA

## Abstract

As online content continues to grow, so does the spread of hate speech. We identify and examine challenges faced by online automatic approaches for hate speech detection in text. Among these difficulties are subtleties in language, differing definitions on what constitutes hate speech, and limitations of data availability for training and testing of these systems. Furthermore, many recent approaches suffer from an interpretability problem—that is, it can be difficult to understand why the systems make the decisions that they do. We propose a multi-view SVM approach that achieves near state-of-the-art performance, while being simpler and producing more easily interpretable decisions than neural methods. We also discuss both technical and practical challenges that remain for this task.

## Introduction

Hate crimes are unfortunately nothing new in society. However, social media and other means of online communication have begun playing a larger role in hate crimes. For instance, suspects in several recent hate-related terror attacks had an extensive social media history of hate-related posts, suggesting that social media contributes to their radicalization [1, 2]. In some cases, social media can play an even more direct role; video footage from the suspect of the 2019 terror attack in Christchurch, New Zealand, was broadcast live on Facebook [2].

Vast online communication forums, including social media, enable users to express themselves freely, at times, anonymously. While the ability to freely express oneself is a human right that should be cherished, inducing and spreading hate towards another group is an abuse of this liberty. For instance, The American Bar Association asserts that in the United States, hate speech is legal and protected by the First Amendment, although not if it directly calls for violence [3]. As such, many online forums such as Facebook, YouTube, and Twitter consider hate speech harmful, and have policies to remove hate speech content [4–6]. Due to the societal concern and how widespread hate speech is becoming on the Internet [7], there is strong motivation to study automatic detection of hate speech. By automating its detection, the spread of hateful content can be reduced.

Detecting hate speech is a challenging task, however. First, there are disagreements in how hate speech should be defined. This means that some content can be considered hate speech to some and not to others, based on their respective definitions. We start by covering competing definitions, focusing on the different aspects that contribute to hate speech. We are by no means, nor can we be, comprehensive as new definitions appear regularly. Our aim is simply to illustrate variances highlighting difficulties that arise from such.

Competing definitions provide challenges for evaluation of hate speech detection systems; existing datasets differ in their definition of hate speech, leading to datastets that are not only from different sources, but also capture different information. This can make it difficult to directly access which aspects of hate speech to identify. We discuss the various datasets available to train and measure the performance of hate speech detection systems in the next section. Nuance and subtleties in language provide further challenges in automatic hate speech identification, again depending on the definition.

Despite differences, some recent approaches found promising results for detecting hate speech in textual content [8–10]. The proposed solutions employ machine learning techniques to classify text as hate speech. One limitation of these approaches is that the decisions they make can be opaque and difficult for humans to interpret *why* the decision was made. This is a practical concern because systems that automatically censor a person’s speech likely need a manual appeal process. To address this problem, we propose a new hate speech classification approach that allows for a better understanding of the decisions and show that it can even outperform existing approaches on some datasets. Some of the existing approaches use external sources, such as a hate speech lexicon, in their systems. This can be effective, but it requires maintaining these sources and keeping them up to date which is a problem in itself. Here, our approach does not rely on external resources and achieves reasonable accuracy. We cover these topics in the following section.

In general, however, there are practical challenges that remain among all systems. For instance, armed with the knowledge that the platforms they use are trying to silence them, those seeking to spread hateful content actively try to find ways to circumvent measures put in place. We cover this topic in more detail in the last section.

In summary, we discuss the challenges and approaches in automatic detection of hate speech, including competing definitions, dataset availability and construction, and existing approaches. We also propose a new approach that in some cases outperforms the state of the art and discuss remaining shortcomings. Ultimately, we conclude the following:

1. Automatic hate speech detection is technically difficult;
2. Some approaches achieve reasonable performance;
3. Specific challenges remain among all solutions;
4. Without societal context, systems cannot generalize sufficiently.

## Defining hate speech

The definition of hate speech is neither universally accepted nor are individual facets of the definition fully agreed upon. Ross, et al. believe that a clear definition of hate speech can help the study of detecting hate speech by making annotating hate speech an easier task, and thus, making the annotations more reliable [11]. However, the line between hate speech and appropriate free expression is blurry, making some wary to give hate speech a precise definition. For instance, the American Bar Association does not give an official definition, but instead asserts that speech that contributes to a criminal act can be punished as part of a hate crime [12]. Similarly, we opt not to propose a specific definition, but instead examine existing definitions to gain insights into what typically constitutes hate speech and what technical challenges the definitions might bring. We summarize leading definitions of hate speech from varying sources, as well as some aspects of the definitions that make the detection of hate speech difficult.

1. Encyclopedia of the American Constitution: “Hate speech is speech that attacks a person or group on the basis of attributes such as race, religion, ethnic origin, national origin, sex, disability, sexual orientation, or gender identity.” [13]
2. Facebook: “We define hate speech as a direct attack on people based on what we call protected characteristics—race, ethnicity, national origin, religious affiliation, sexual orientation, caste, sex, gender, gender identity, and serious disease or disability. We also provide some protections for immigration status. We define attack as violent or dehumanizing speech, statements of inferiority, or calls for exclusion or segregation.” [4]
3. Twitter: “Hateful conduct: You may not promote violence against or directly attack or threaten other people on the basis of race, ethnicity, national origin, sexual orientation, gender, gender identity, religious affiliation, age, disability, or serious disease.” [6]
4. Davidson et al.: “Language that is used to expresses hatred towards a targeted group or is intended to be derogatory, to humiliate, or to insult the members of the group.” [9]
5. de Gilbert et al.: “Hate speech is a deliberate attack directed towards a specific group of people motivated by aspects of the group’s identity.” [14]
6. Fortuna et al. “Hate speech is language that attacks or diminishes, that incites violence or hate against groups, based on specific characteristics such as physical appearance, religion, descent, national or ethnic origin, sexual orientation, gender identity or other, and it can occur with different linguistic styles, even in subtle forms or when humour is used.” [8]. This definition is based on their analysis of various definitions.

It is notable that in some of the definitions above, a necessary condition is that it is directed to a group. This differs from the Encyclopedia of the American Constitution definition, where an attack on an individual can be considered hate speech. A common theme among the definitions is that the attack is based on some aspect of the group or peoples identity. While in de Gilbert’s definition the identity itself is left vague, some of the other definitions provide specific identity characteristics. In particular, protected characteristics are aspects of the Davidson et al. and Facebook definitions. Fortuna et al.’s definition specifically calls out variations in language style and subtleties. This can be challenging, and goes beyond what conventional text-based classification approaches are able to capture.

Fortuna et al.’s definition is based on an analysis of the following characteristics from other definitions [8]:

1. Hate speech is to incite violence or hate
2. Hate speech is to attack or diminish
3. Hate speech has specific targets
4. Whether humor can be considered hate speech

A particular problem not covered by many definitions relate to factual statements. For example, “Jews are swine” is clearly hate speech by most definitions (it is a statement of inferiority), but “Many Jews are lawyers” is not. In the latter case, to determine whether each statement is hate speech, we would need to check whether the statement is factual or not using external sources. This type of hate speech is difficult because it relates to real-world fact verification—another difficult task [15]. More so, to evaluate validity, we would initially need to define precise word interpretations, namely, is “many” an absolute number or by relative percentage of the population, further complicating the verification.

Another issue that arises in the definition of hate speech is the potential praising of a group that is hateful. For example, praising the KKK is hate speech, however praising another group can clearly be non-hate speech. In this case it is important to know what groups are hate groups and what exactly is being praised about the group as some praising is undoubtedly, and unfortunately, true. For example, the Nazis were very efficient in terms of their “Final Solution”. Thus, praise processing alone is, at times, difficult.

## Datasets

Collecting and annotating data for the training of automatic classifiers to detect hate speech is challenging. Specifically, identifying and agreeing whether specific text is hate speech is difficult, as per previously mentioned, there is no universal definition of hate speech. Ross, et al. studied the reliability of hate speech annotations and suggest that annotators are unreliable [11]. Agreement between annotators, measured using Krippendorff’s *α*, was very low (up to 0.29). However, they compared annotations based on the Twitter definition, versus annotations based on their own opinions and found a strong correlation.

Furthermore, social media platforms are a hotbed for hate speech, yet many have very strict data usage and distribution policies. This results in a relatively small number of datasets available to the public to study, with most coming from Twitter (which has a more lenient data usage policy). While the Twitter resources are valuable, their general applicability is limited due to the unique genre of Twitter posts; the character limitation results in terse, short-form text. In contrast, posts from other platforms are typically longer and can be part of a larger discussion on a specific topic. This provides additional context that can affect the meaning of the text.

Another challenge is that there simply are not many publicly-available, curated datasets that identify hateful, aggressive, and insulting text. A representative sampling of available training and evaluation public datasets is shown in Table 1:

- **HatebaseTwitter** [9]. One Twitter dataset is a set of 24,802 tweets provided by Davidson, et al [9]. Their procedure for creating the dataset was as follows. First they took a hate speech lexicon from Hatebase [16] and searched for tweets containing these terms, resulting in a set of tweets from about 33,000 users. Next they took a timeline from all these users resulting in a set of roughly 85 million Tweets. From the set of about 85 million tweets, they took a random sample, of 25k tweets, that contained terms from the lexicon. Via crowdsourcing, they annotated each tweet as hate speech, offensive (but not hate speech), or neither hate speech nor offensive. If the agreement between annotators was too low, the tweet was excluded from the set. A commonly-used subset of this dataset is also available, containing 14,510 tweets.
- **WaseemA** [17]. Waseem and Hovy also provide a dataset from Twitter, consisting of 16,914 tweets labeled as racist, sexist, or neither [17]. They first created a corpus of about 136,000 tweets that contain slurs and terms related to religious, sexual, gender, and ethnic minorities. From this corpus, the authors themselves annotated (labeled) 16,914 tweets and had a gender studies major review the annotations.
- **WaseemB** [18]. In a second paper, Waseem creates another dataset by sampling a new set of tweets from the 136,000 tweet corpus [18]. In this collection, Waseem recruited feminists and anti-racism activists along with crowdsourcing for the annotation of the tweets. The labels therein are racist, sexist, neither or both.
- **Stormfront** [14]. de Gilbert, et al. provide a dataset from posts from a white supremacist forum, Stormfront [14]. They annotate the posts at sentence level resulting in 10,568 sentences labeled with Hate, NoHate, Relation, or Skip. Hate and NoHate labels indicate presence or lack thereof, respectively, of hate speech in each sentence. The label “Relation” indicates that the sentence is hate speech when it is combined with the sentences around it. Finally, the label “skip” is for sentences that are non-English or not containing information related to hate or non-hate speech. They also capture the amount of context (i.e., previous sentences) that an annotator used to classify the text.
- **TRAC** [19]. The 2018 Workshop on Trolling, Aggression, and Cyberbullying (TRAC) hosted a shared task focused on detecting aggressive text in both English and Hindi [19]. Aggressive text is often a component of hate speech. The dataset from this task is available to the public and contains 15,869 Facebook comments labeled as overtly aggressive, covertly aggressive, or non-aggressive. There is also a small Twitter dataset, consisting of 1,253 tweets, which has the same labels.
- **HatEval** [20]. This dataset is from SemEval 2019 (Task 5) for competition on multilingual detection of hate targeting to women and immigrants in tweets [20]. It consists of several sets of labels. The first indicates whether the tweet expresses hate towards women or immigrants, the second, whether the tweet is aggressive, and the third, whether the tweet is directed at an individual or an entire group. Note that targeting an individual is not necessarily considered hate speech by all definitions.
- **Kaggle** [21] Kaggle.com hosted a shared task on detecting insulting comments [21]. The dataset consists of 8,832 social media comments labeled as insulting or not insulting. While not necessarily hate speech, insulting text may indicate hate speech.
- **GermanTwitter** [11]. As part of their study of annotator reliability, Ross, et al. created a Twitter dataset in German for the European refugee crisis [11]. It consists of 541 tweets in German, labeled as expressing hate or not.

**Table 1:** Hate-related dataset characteristics.

Dataset	Labels and percents in dataset	Origin Source	Language
HatebaseTwitter [9]	Hate 5% Offensive 76% Neither 17%	Twitter	English
WaseemA [17]	Racism 12% Sexism 20% Neither 68%	Twitter	English
WaseemB [18]	Racism1 1% Sexism 13% Neither 84% Both 1%	Twitter	English
Stormfront [14]	Hate 11% Not Hate 86% Relation 2% Skip 1%	Online Forum	English
TRAC (Facebook) [19]	Non-aggressive 69% Overtly agg. 16% Covertly agg. 16%	Facebook	English & Hindi
TRAC (Twitter) [19]	Non-aggressive 38% Overtly agg. 29% Covertly agg. 33%	Twitter	English & Hindi
HatEval [20]	Hate 43% / Not Hate 57% Agg. / Not agg. roup / Individual	Twitter	English & Spanish
Kaggle [21]	Insulting 26% Not Insulting 74%	Twitter	English
GermanTwitter (Expert 1 annotation) [11]	Hate 23% Not Hate 77%	Twitter	German

Note that these datasets vary considerably in their size, scope, characteristics of the data annotated, and characteristics of hate speech considered. The most common source of text is Twitter, which consists of short-form online posts. While the Twitter datasets do capture a wide variety of hate speech aspects in several different languages such as attacking different groups, the construction process including the filtering and sampling methods introduce uncontrolled factors for analyzing the corpora. Furthermore, corpora constructed from social media and websites other than Twitter are rare, making analysis of hate speech difficult to cover the entire landscape.

There is also the issue of imbalance in the number of hate and not hate texts within datasets. On a platform such as Twitter, hate speech occurs at a very low rate compared to non-hate speech. Although datasets reflect this imbalance to an extent, they do not map the actual percentage due to training needs. For example, in the WaseemA dataset [17], 20% of the tweets were labelled sexist, 11.7% racist, and 68.3% neither. In this case, there is still an imbalance in the number of sexist, racist, or neither tweets, but it may not be as imbalanced as expected on Twitter.

## Automatic approaches for hate speech detection

Most social media platforms have established user rules that prohibit hate speech; enforcing these rules, however, requires copious manual labor to review every report. Some platforms, such as Facebook, recently increased the number of content moderators. Automatic tools and approaches could accelerate the reviewing process or allocate the human resource to the posts that require close human examination. In this section, we overview automatic approaches for hate speech detection from text.

### Keyword-based approaches

A basic approach for identifying hate speech is using a keyword-based approach. By using an ontology or dictionary, text that contain potentially hateful keywords are identified. For instance, Hatebase [16] maintains a database of derogatory terms for many groups across 95 languages. Such well-maintained resources are valuable, as terminology changes over time. However, as we observed in our study of the definitions of hate speech, simply using a hateful slur is not necessarily enough to constitute hate speech.

Keyword-based approaches are fast and straightforward to understand. However, they have severe limitations. Detecting only racial slurs would result in a highly precise system but with low recall where precision is the percentage of relevant from the set detected and recall is the percent of relevant from within the global population. In other words, a system that relies chiefly on keywords would not identify hateful content that does not use these terms. In contrast, including terms that could but are not always hateful (e.g., “trash”, “swine”, etc.) would create too many false alarms, increasing recall at the expense of precision.

Furthermore, keyword-based approaches cannot identify hate speech that does not have any hateful keywords (e.g., figurative or nuanced language). Slang such as “build that wall” literally means constructing a physical barrier (wall). However, with the political context, some interpret this is a condemnation of some immigrates in the United States.

### Source metadata

Additional information from social media can help further understand the characteristics of the posts and potentially lead to a better identification approach. Information such as demographics of the posting user, location, timestamp, or even social engagement on the platform can all give further understanding of the post in different granularity.

However, this information is not often readily available to external researchers as publishing data with sensitive user information raises privacy issues. External researchers might only have part or even none of the user information. Thus, they possibly solve the wrong puzzle or learn based on wrong knowledge from the data. For instance, a system trained on these data might naturally bias towards flagging content by certain users or groups as hate speech based on incidental dataset characteristics.

Using user information potentially raises some ethical issues. Models or systems might be biased against certain users and frequently flag their posts as hateful even if some of them are not. Similarly, relying too much on demographic information could miss posts from users who do not typically post hateful content. Flagging posts as hate based on user statistics could create a chilling effect on the platform and eventually limit freedom of speech.

### Machine learning classifiers

Machine learning models take samples of labeled text to produce a classifier that is able to detect the hate speech based on labels annotated by content reviewers. Various models were proposed and proved successful in the past. We describe a selection of open-sourced systems presented in the recent research.

#### Content preprocessing and feature selection

To identify or classify user-generated content, text features indicating hate must be extracted. Obvious features are individual words or phrases (n-grams, i.e., sequence of *n* consecutive words). To improve the matching of features, words can be stemmed to obtain only the root removing morphological differences. Metaphore processing, e.g., Neuman, et. al. [22], likewise can extract features.

The bag-of-words assumption is commonly used in text categorization. Under this assumption, a post is represented simply as a set of words or n-grams without any ordering. This assumption certainly omits an important aspect of languages but nevertheless proved powerful in numerous tasks. In this setting, there are various ways to assign weights to the terms that may be more important, such as TF-IDF [23]. For a general information retrieval review, see [24].

Besides distributional features, word embeddings, i.e., assigning a vector to a word, such as word2vec [25], are common when applying deep learning methods in natural language processing and text mining [26, 27]. Some deep learning architectures, such as recurrent and transformer neural networks, challenge the bag-of-words assumption by modeling the ordering of the words by processing over a sequence of word embeddings [28].

#### Hate speech detection approaches and baselines

**Naïve Bayes, Support Vector Machine and Logistic Regression**. These models are commonly used in text categorization. Naïve Bayes models label probabilities directly with the assumption that the features do not interact with one another. Support Vector Machines (SVM) and Logistic Regression are linear classifiers that predict classes based on a combination of scores for each feature. Open-source implementations of the these models exist, for instance in the well-known Python machine learning package *sci-kit learn* [29].

**Davidson, et al**. [9] Davidson, et al. proposed a state-of-the-art feature-based classification model that incorporates distributional TF-IDF features, part-of-speech tags, and other linguistic features using support vector machines. The incorporation of these linguistic features helps identify hate speech by distinguishing between different usages of the terms, but still suffers from some subtleties, such as when typically offensive terms are used in a positive sense (e.g., *queer* in “He’s a damn good actor. As a gay man, it’s awesome to see an openly queer actor given the lead role for a major film.”, from HatebaseTwitter dataset [9]).

**Neural Ensemble** [10]. Zimmerman, et al. propose an ensemble approach, which combines the decisions of ten convolutional neural networks with different weight initializations [10]. Their network structure is similar to the one proposed by [30], with convolutions of length 3 pooled over the entire document length. The results of each model are combined by averaging the scores, akin to [31].

**FastText** [32]. FastText is an efficient classification model proposed by researchers in Facebook. The model produces embeddings of character n-grams and provides predictions of the example based on the embeddings. Over time, this model has become a strong baseline for many text categorization tasks.

**BERT** [26]. BERT is a recent transformer-based pre-trained contextualized embedding model extendable to a classification model with an additional output layer. It achieves state-of-the-art performance in text classification, question answering, and language inference without substantial task-specific modifications. When we experiment with BERT, we add a linear layer atop the classification token (as suggested by [26]), and test all suggested tuning hyperparameters.

**C-GRU** [33]. C-GRU, a Convolution-GRU Based Deep Neural Network proposed by Zhang, et al., combines convolutional neural networks (CNN) and gated recurrent networks (GRU) to detect hate speech on Twitter. They conduct several evaluations on publicly available Twitter datasets demonstrating their ability to capture word sequence and order in short text. Note, in the HatebaseTwitter [9] dataset, they treat both Hate and Offensive as Hate resulting in binary label instead of its original multi-class label. In our evaluation, we use the original multi-class labels where different model evaluation results are expected.

### Our proposed classifier: Multi-view SVM

We propose a multi-view SVM model for the classification of hate speech. It applies a multiple-view stacked Support Vector Machine (mSVM) [34]. Each type of feature (e.g., a word TF-IDF unigram) is fitted with an individual Linear SVM classifier (inverse regularization constant *C* = 0.1), creating a *view-classifier* for those features. We further combine the view classifiers with another Linear SVM (*C* = 0.1) to produce a *meta-classifier*. The features used in the meta-classifier are the predicted probability of each label by each view-classifier. That is, if we have 5 types of features (e.g., character unigram to 5-gram) and 2 classes of labels, 10 features would serve as input into the meta-classifier.

Combining machine learning classifiers is not a new concept [35]. Previous efforts have shown that combining SVM with different classifiers provides improvements to various data mining tasks and text classification [36, 37]. Combining multiple SVMs (mSVMs) has also been proven to be an effective approach in image processing tasks for reducing the large dimensionality problem [38].

However, applying multiple SVMs to identify hate speech expands the domain of use for such classification beyond that previously explored. Multi-view learning is known for capturing different *views* of the data [34]. In the context of hate speech detection, incorporating different views captures differing aspects of hate speech within the classification process. Instead of combining all features into a single feature vector, each view-classifier learns to classify the sentence based on only one type of feature. This allows the view-classifiers to pick up different aspects of the pattern individually.

Integrating all feature types in one model, by regularization, risks the masking of relatively weak but key signals. For example, “yellow” and “people” individually would appear more times than “yellow people” combined; posts having these terms individually are unlikely to be hate. However, “yellow people” is likely hate speech (especially when other hate speech aspects are present), but the signal might be rare in the collection, and therefore, is likely masked by the regularization if all features are combined together. In this case, mSVM is able to pick up this feature in one of the view-classifiers, where there are fewer parameters.

Furthermore, this model offers the opportunity to interpret the model so as to identify which view-classifier contributes most through the meta-classifier provides human intuition for the classification. The view-classifier contributing most to the final decision identifies key vocabulary (features) resulting in a hate speech label. This contrasts with well-performing neural models, which are often opaque and difficult to understand [10, 39, 40]. Even state-of-the-art methods that employ self-attention (e.g., BERT [26]) suffer from considerable noise that vastly reduces interpretability.

### Experimental setup

Using multiple hate speech datasets, we evaluated the accuracy of existing as well as our hate speech detection approaches.

#### Data preprocessing and features

For simplicity and generality, preprocessing and feature identification is intentionally minimal. For pre-processing, we apply case-folding, tokenization, and punctuation removal (while keeping emoji). For features, we simply extract word TF-IDF from unigram to 5-gram and character N-gram counts from unigram to 5-gram.

#### Datasets

We evaluate the approach on the Stormfront [14], TRAC [19], HatEval, and HatebaseTwitter [9] datasets previously described. These datasets provide a variety of hate speech definitions and aspects (including multiple types of aggression), and multiple types of online content (including online forums, Facebook, and Twitter content). For Stormfront, we use the balanced train/test split proposed in [14], with a random selection of 10% of the training set held out as validation data. For the TRAC dataset, we use the English Facebook training, validation, and test splits provided by [19]. For HatEval, we use a split of the training set for validation and use the official validation dataset for testing because the official test set is not public. Finally, for the HatebaseTwitter dataset [9], we use the standard train-validation-test split provided by [9].

#### Evaluation

We evaluate the performance of each approach using accuracy and macro-averaged *F*_1_ score. There are not a consensus in literature about which evaluation metrics to use. However, we believe that focusing on both accuracy and macro-*F*_1_ offers good insights into the relative strengths and weaknesses of each approach.

### Experimental results

We report the highest score of the approaches described above on each dataset in Table 2. Complete evaluation results are available in supporting document S1 Table (including accuracy breakdown by label).

**Table 2:** Hate speech classification results. The top two approaches on each dataset are reported.

Dataset	Model	Accuracy	Macro *F*_1_
Stormfront	BERT	0.8201	0.8201
	mSVM (ours)	0.8033	0.8031
TRAC (Facebook)	mSVM (ours)	0.6121	0.5368
	BERT	0.5809	0.5234
HatebaseTwitter	Neural Ensemble	0.9217	0.9118
	BERT	0.9209	0.8917
HatEval	BERT	0.7480	0.7452
	Neural Ensemble	0.7470	0.7481

In the Stormfront and TRAC datasets, our proposed approach provides state-of-the-art or competitive results for hate speech detection. On Stormfront, the mSVM model achieves 80% accuracy in detecting hate speech, which is a 7% improvement from the best published prior work (which achieved 73% accuracy). BERT performs 2% better than our approach, but the interpretability of the decisions the BERT model made are difficult to explain.

On the TRAC dataset, our mSVM approach achieves 53.68% macro *F*_1_ score. Note that through optimization on the validation set, we found that using TF-IDF weights for character N-grams works better on Facebook dataset, so we report results using those TF-IDF instead of raw counts. This outperforms all other approaches we experimented with, including the strong BERT system. We also compared our approach to the other systems that participated in the shared task [19], and observed that we outperform them as well in terms of the metric they reported (weighted F-score) by 1.34% or higher. This is particularly impressive because our approach outperformed systems which rely on external datasets and data augmentation strategies.

Our approach outperformed the top-ranked ensemble [41] method by 3.96% in terms of accuracy and 2.41% in terms of *F*_1_. This indicates that mSVM learns from different aspects and preserves more signals as compared to a simple ensemble method that uses all features for each first-level classifier. BERT achieved 3% lower in terms of accuracy and 1% lower in terms of *F*_1_ than our proposed method and still provided minimal interpretability, demonstrating that forgoing interpretability does not necessarily provide higher accuracy. For HatEval and HatebaseTwitter, the neural ensemble approach outperforms our method suggesting that neural approaches are better suited for Twitter data than mSVM-based solution. Previous works reported various metrics, e.g. a support-weighted *F*_1_ in Davidson, et. al. [9], making comparison between models difficult. We report macro *F*_1_ to mitigate the effect of imbalance between classes, which is an effect that has been baked in during the construction of the datasets. For a fair and complete comparison between the systems, we execute the systems from the previous works and calculate macro *F*_1_ on the datasets reported in this study. The previous best performance on the Stormfront dataset used a recurrent neural network to achieve an accuracy of 0.73 [14]; our approach easily outperforms this method. On the TRAC dataset, others reported a weighted *F*_1_ performance of 0.6425 using a recurrent neural network, without reporting accuracy or macro-averaged *F*_1_ [19, 42]. On HatebaseTweitter, others reported a macro *F*_1_ score of 0.94 [33], but this is achieved by combining the hate and offensive categories, greatly simplifying the task.

In S1 Table, we observe that for most datasets and approaches, the accuracy is biased towards the majority class in the training data. This suggests the need for datasets that are more representative of real data distributions for future evaluation.

Considering the above mixed in terms of dominance evaluation results, given potential ethical concerns related to hate speech detection, we err on the side of caution and opt for interpretability over uncertain improvements on the evaluation metrics.

#### Interpretation of mSVM

We analyzed the top features of the mSVM classifier on the Stormfront dataset. The meta-classifier weights character 4-grams and word unigrams as the highest contributors to the overall score. 4-grams such as “jew”, “ape”, “mud”, “egro” are among the strongest signals of being hate. (Note that whitespace contributes to character 4-grams.) This category appears to capture the aspect of a group’s identity. Word unigrams such as “invasion” and “violence” contribute highly to hate classification, and appear to capture the attack aspect. The top word unigrams, 2-grams and 3-grams from the view-classifier results of each dataset are in S3 Table. We found that the accuracy of all view-classifiers is at least two percent lower than the meta-classifier. The full comparison between view-classifier and meta-classifier results are given in supplementary information S2 Table. We also found that, although three other view-classifiers outperform the word unigram model, the meta-classifier still weights its score higher than those models, further suggesting that it captures a different hate speech aspect.

#### Interpretation of BERT

**Fig 1:**
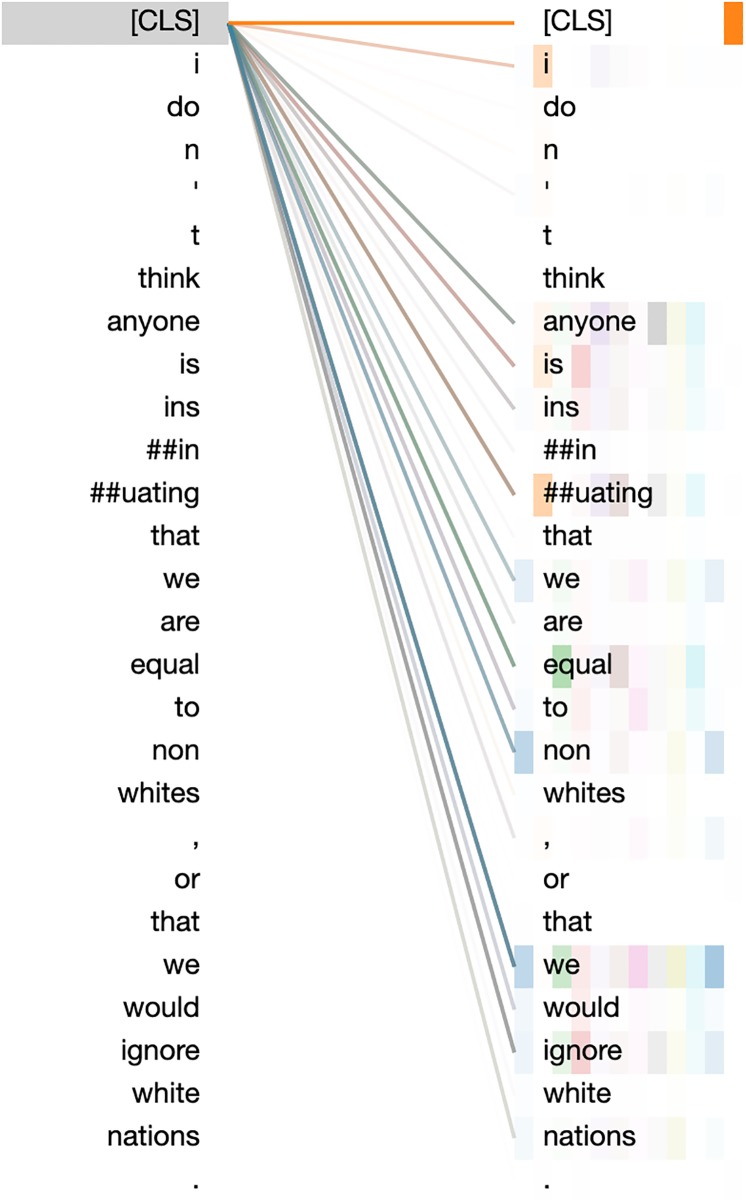
Self-attention weights for the classification token of the trained BERT model for a sample post. Each color represents a different attention head, and the lightness of the color represents the amount of attention. For instance, the figure indicates that nearly all attention heads focus heavily on the term ‘we’.

Because the BERT model employs a self-attention mechanism, one can visualize the terms that the model relies most upon for classification purposes. We present attention visualizations from BertViz [43] for the trained BERT model on the mis-classified forum post *“I don’t think anyone is insinuating that we are equal to non whites, or that we would ignore white nations.”* (this post does not satisfy the authors’ conditions for hate speech, but the BERT model classified it as hateful). We present an detailed attention weights for all 12 attention heads of the classification token on layer 11 in Fig 1. Despite appearing to be the most informative layer, we observe that Layer 11 does not provide a clear answer to why the model labeled the post as hateful; the attention is distributed among most words in the sentence, and many of the weights with the most attention do not appear to be informative (e.g., *we*). When investigating the other layers (overview given in S1 Fig in the supplementary information) and other posts, we similarly do not see strong trends that would enable interpretability. This demonstrates the limitation of using deep neural models—even those with claims of interpretability—when trying to interpret the decisions made. These observations are in line with prior work that has found attention signals to be noisy and not necessarily indicative of term importance [39, 40]. While our approach can be combined with neural models, it would come at the expense of increased model complexity and reduced interpretability.

#### Error analysis

To gain a better understanding of our mSVM classifier’s mistakes, we qualitatively analyze its false positive (FP) and false negative (FN) samples on the Stormfront dataset. We categorized the misclassified posts based on their mutual linguistic features, semantic features, and length. 41% of the posts misclassified as not hate needed surrounding context to understand that the post is hate speech. 7% of the FN were implicit hate, making it difficult to classify, such as “*Indeed, I haven’t seen or heard machines raping or robbing people in the streets of Stockholm yet, non-european immigrants however…*”. Furthermore, given that the inter-annotator agreement is not perfect in the dataset [14] (prior work shows that high inter-annotator agreement for hate speech is difficult to achieve [11, 44]), we analyzed some borderline cases with the definition of hate speech used for annotation. When manually re-assessing the misclassified posts, we found that the gold label of the 17% of the FN and 10% of the FP posts do not match our interpretation of the post content. Another major problem is with posts that are aggressive but do not meet the necessary conditions to be considered hate speech. These constitute 16% of the FP. Finally, short posts (6 or fewer terms, representing less than 3% of hate speech sentences found in the dataset) increased FP as well, occurring 7% of the time. The remaining misclassified posts were miscellaneous cases including posts that are sarcastic or metaphoric.

## Shortcomings and future work

A challenge faced by automatic hate speech detection systems is the changing of attitudes towards topics over time and historical context. Consider the following excerpt of a Facebook post:

“…The merciless Indian Savages, whose known rule of warfare, is an undistinguished destruction of all ages, sexes and conditions…”

Intuition suggests that this is hate speech; it refers to Native Americans as “merciless Indian savages”, and dehumanizes them by suggesting that they are inferior. Indeed, the text satisfies conditions used in most definitions of hate speech. However, this text is actually a quote from the Declaration of Independence. Given the historical context of the text, the user who posted it may not have intended the hate speech result, but instead meant to quote the historical document for other purposes. This shows that user intent and context play an important role in hate speech identification.

As another example, consider the phrase “the Nazi organization was great.” This would be considered hate speech because it shows support for a hate group. However, “the Nazi’s organization was great” isn’t supporting their ideals but instead commenting on how well the group was organized. In some contexts, this might not be considered hate speech, e.g., if the author was comparing organizational effectiveness over time. The difference in these two phrases is subtle, but could be enough to make the difference between hate speech or not.

Another remaining challenge is that automatic hate speech detection is a closed-loop system; individuals are aware that it is happening, and actively try to evade detection. For instance, online platforms removed hateful posts from the suspect in the recent New Zealand terrorist attack (albeit manually), and implemented rules to automatically remove the content when re-posted by others [2]. Users who desired to spread the hateful messages quickly found ways to circumvent these measures by, for instance, posting the content as images containing the text, rather than the text itself. Although optical character recognition can be employed to solve the particular problem, this further demonstrates the difficulty of hate speech detection going forward. It will be a constant battle between those trying to spread hateful content and those trying to block it.

## Conclusion

As hate speech continues to be a societal problem, the need for automatic hate speech detection systems becomes more apparent. We presented the current approaches for this task as well as a new system that achieves reasonable accuracy. We also proposed a new approach that can outperform existing systems at this task, with the added benefit of improved interpretability. Given all the challenges that remain, there is a need for more research on this problem, including both technical and practical matters.

## Supporting information

S1 Table Full comparison of hate speech classifiers. (PDF)

S2 Table Full comparison of view classifiers in mSVM. (PDF)

S3 Table Top 10 weighted vocabularies learned by Word-level view classifier. This list has been sanitized.
(PDF)

S1 Fig Visualization of self-attention weights for the forum BERT model. All layers and attention heads for the sentence “*I don’t think anyone is insinuating that we are equal to non whites, or that we would ignore white nations.*” are included. Darker lines indicate stronger attention between terms. The first token is the special classification token.
(PNG)
