# Supplementary material for: Hate speech detection: Challenges and solutions
Source: PLoS One. 2019 Aug 20;14(8):e0221152. doi: 10.1371/journal.pone.0221152 (PMC6701757; doi:10.1371/journal.pone.0221152)
Supplement: S1 Table — (PDF) [file pone.0221152.s001.pdf]

**Stormfront**

| Method | Accuracy All | Accuracy Hate | Accuracy Not Hate | Macro $F_1$ |
|---|---|---|---|---|
| Naïve Bayes | 0.6423 | 0.6164 | 0.6828 | 0.6378 |
| SVM | 0.7469 | 0.7438 | 0.7500 | 0.7469 |
| Logistic Regression | 0.7218 | 0.7155 | 0.7280 | 0.7217 |
| FastText | 0.6506 | 0.6406 | 0.6622 | 0.6502 |
| Davidson et al. [9] | 0.7364 | 0.7344 | 0.7384 | 0.7364 |
| Neural Ensemble [10] | 0.8033 | 0.7736 | 0.8404 | 0.8027 |
| mSVM (ours) | 0.8033 | 0.8251 | 0.7843 | 0.8031 |
| BERT [26] | 0.8201 | 0.8255 | 0.8148 | 0.8201 |
| C-GRU [33] | 0.6297 | 0.5969 | 0.6962 | 0.6188 |

**HatEval**

| Method | Accuracy All | Accuracy Hate | Accuracy Not Hate | Macro $F_1$ |
|---|---|---|---|---|
| Naïve Bayes | 0.6800 | 0.6253 | 0.7208 | 0.6730 |
| SVM | 0.7190 | 0.6594 | 0.7694 | 0.7152 |
| Logistic Regression | 0.7340 | 0.6801 | 0.7776 | 0.7297 |
| FastText | 0.7380 | 0.6846 | 0.7812 | 0.7338 |
| Davidson et al. [9] | 0.7390 | 0.6869 | 0.7806 | 0.7346 |
| Neural Ensemble [10] | 0.7470 | 0.6867 | 0.7996 | 0.7441 |
| mSVM (ours) | 0.7590 | 0.7143 | 0.7933 | 0.7543 |
| BERT [26] | 0.7480 | 0.6866 | 0.8023 | 0.7452 |
| C-GRU [33] | 0.6670 | 0.6399 | 0.6802 | 0.6471 |

**TRAC(Facebook)**

| Method | Accuracy All | Accuracy NAG[1] | Accuracy CAG[2] | Accuracy OAG[3] | Macro $F_1$ |
|---|---|---|---|---|---|
| Naïve Bayes | 0.4758 | 0.8306 | 0.2111 | 0.2640 | 0.4080 |
| SVM | 0.5714 | 0.8326 | 0.2444 | 0.4762 | 0.5050 |
| Logistic Regression | 0.5556 | 0.8450 | 0.2421 | 0.4438 | 0.5001 |
| FastText | 0.5626 | 0.8326 | 0.2246 | 0.4800 | 0.4879 |
| Davidson et al. [9] | 0.5604 | 0.8428 | 0.2259 | 0.4235 | 0.4875 |
| Neural Ensemble [10] | 0.5358 | 0.8575 | 0.2306 | 0.4647 | 0.4945 |
| mSVM (ours) | 0.6121 | 0.8479 | 0.2589 | 0.5202 | 0.5368 |
| BERT [26] | 0.5809 | 0.8538 | 0.2516 | 0.4881 | 0.5234 |
| C-GRU [33] | 0.4769 | 0.7436 | 0.1818 | 0.2156 | 0.3696 |

[1] not aggressive [2] covertly aggressive [3] overtly aggressive

**HatebaseTwitter**

| Method | Accuracy | | | | Macro |
|---|---|---|---|---|---|
| | All | Hate[1] | Off.[2] | N[3] | $F_1$ |
| Naïve Bayes | 0.8297 | 0.2571 | 0.8418 | 0.7940 | 0.5138 |
| SVM | 0.9092 | 0.5429 | 0.9326 | 0.8282 | 0.6788 |
| Logistic Regression | 0.9149 | 0.5000 | 0.9409 | 0.8356 | 0.6914 |
| FastText | 0.9068 | 0.5100 | 0.9346 | 0.8220 | 0.6930 |
| Davidson et al. [9] | 0.9007 | 0.6098 | 0.9270 | 0.8033 | 0.6877 |
| Neural Ensemble [10] | 0.9213 | 0.5179 | 0.9453 | 0.8628 | 0.7218 |
| mSVM (ours) | 0.9108 | 0.4961 | 0.9585 | 0.8251 | 0.7704 |
| BERT [26] | 0.9209 | 0.4857 | 0.9499 | 0.8917 | 0.7609 |
| C-GRU [33] | 0.8588 | 0.5556 | 0.9065 | 0.6550 | 0.5651 |

[1] hate speech [2] offensive language [3] neither
