# Supplementary material for: Hate speech detection: Challenges and solutions
Source: PLoS One. 2019 Aug 20;14(8):e0221152. doi: 10.1371/journal.pone.0221152 (PMC6701757; doi:10.1371/journal.pone.0221152)
Supplement: S2 Table — (PDF) [file pone.0221152.s002.pdf]

Stromfront

	Accuracy All	Accuracy Hate	Accuracy Not Hate	Macro F_1
Full model	0.8033	0.8251	0.7843	0.8031
Word-level View				
Unigram	0.7175	0.7203	0.7149	0.7156
2-gram	0.5188	0.5111	0.5634	0.4510
3-gram	0.5021	0.6000	0.5011	0.3452
4-gram	0.5000	0.5000	0.5000	0.3370
5-gram	0.5021	1.0000	0.5010	0.3380
Character-level View				
Unigram	0.6213	0.5929	0.6747	0.6123
2-gram	0.7050	0.6899	0.7227	0.7046
3-gram	0.7531	0.7266	0.7867	0.7523
4-gram	0.7678	0.7520	0.7857	0.7676
5-gram	0.7615	0.7470	0.7778	0.7613

HatEval

	Accuracy All	Accuracy Hate	Accuracy Not Hate	Macro F_1
Full model	0.7590	0.7143	0.7933	0.7543
Word-level View				
Unigram	0.7490	0.7222	0.7666	0.7409
2-gram	0.7040	0.6756	0.7209	0.6917
3-gram	0.5830	0.7083	0.5800	0.4031
4-gram	0.5790	0.7143	0.5771	0.3877
5-gram	0.5770	0.8333	0.5755	0.3766
Character-level View				
Unigram	0.6840	0.6282	0.7266	0.6777
2-gram	0.7060	0.6572	0.7418	0.6992
3-gram	0.7360	0.6983	0.7623	0.7289
4-gram	0.7520	0.7243	0.7704	0.7443
5-gram	0.7500	0.7263	0.7652	0.7414

TRAC (Facebook)

	Accuracy				Macro
	All	NAG[1]	CAG[2]	OAG[3]	F_1
Full model	0.6121	0.8479	0.2589	0.5202	0.5368
Word-level View					
Unigram	0.5813	0.8063	0.2357	0.4766	0.4828
2-gram	0.6538	0.7030	0.2500	0.4000	0.3473
3-gram	0.6890	0.6967	0.5000	0.1429	0.3107
4-gram	0.6879	0.6939	0.5000	0.1429	0.2945
5-gram	0.6846	0.6904	0.4000	0.1429	0.2848
Character-level View					
Unigram	0.5615	0.7543	0.2127	0.4706	0.3727
2-gram	0.5593	0.7992	0.2283	0.4151	0.4573
3-gram	0.5714	0.8207	0.2381	0.4924	0.4999
4-gram	0.5945	0.8285	0.2616	0.5077	0.5192
5-gram	0.5824	0.8172	0.2476	0.4826	0.4956

[1] not aggressive [2] covertly aggressive [3] overtly aggressive

HatebaseTwitter

	Accuracy				Macro
	All	Hate[1]	Off.[2]	N[3]	F_1
Full model	0.9108	0.4961	0.9585	0.8251	0.7704
Word-level View					
Unigram	0.8955	0.5882	0.9080	0.8400	0.6307
2-gram	0.7926	1.0000	0.7919	0.9091	0.3210
3-gram	0.7885	0.0000	0.7885	1.0000	0.2956
4-gram	0.7885	0.0000	0.7885	1.0000	0.2956
5-gram	0.7885	0.0000	0.7885	1.0000	0.2956
Character-level View					
Unigram	0.7978	0.5000	0.8067	0.5833	0.3766
2-gram	0.8688	0.6250	0.8870	0.7663	0.5893
3-gram	0.8947	0.6250	0.9081	0.8311	0.6277
4-gram	0.8910	0.6667	0.9010	0.8380	0.6065
5-gram	0.8874	0.6250	0.8919	0.8642	0.5953

[1] hate speech [2] offensive language [3] neither
