# Supplementary material for: Hate speech detection: Challenges and solutions
Source: PLoS One. 2019 Aug 20;14(8):e0221152. doi: 10.1371/journal.pone.0221152 (PMC6701757; doi:10.1371/journal.pone.0221152)
Supplement: S3 Table — This list has been sanitized. (PDF) [file pone.0221152.s003.pdf]

Stormfront

	Hate	Not Hate
Unigram	jews	youtube
	black	thank
	n*gro	welcome
	scum	pm
	race	check
	white	idea
	ape	sf
	africa	link
	asian	happy
	place	join
2-gram	non white	welcome sf
	race mixing	watch tv
	crippin n*gga	sounds like
	black people	thank posting
	like blacks	good luck
	white woman	years ago
	race traitors	like minded
	white countries	home school
	blacks asians	yankee jim
3-gram	homosexuals stay closet	like minded people
	way advantage state	love big dog
	liberals care diversity	treading ice jimmy
	reality money charade	like comment link
	pakis forcing culture	readily happy welcome
	think exempt rules	sir thomas lawrence
	i'm winnipeg cesspool	30 years ago
	wonder races achieve	think write book
	maximum resistance zog	hope talk later
	white genocide project	camellia idea great

HatEval

	Hate	Not Hate
Unigram	h*e	immigrant
	nodaca	men
	wh*re	ram
	maga	k*nt
	buildthewall	son
	b*tches	calling
	illegal	h*
	womensuck	stand
	buildthatwall	rohingya
	b*tch	thank
2-gram	b*tch f*ck	immigrant children
	b*tch h*e	immigrant families
	ass b*tch	anti immigrant
	nodaca noamnesty	men
	women stupid	migrants https
	trump maga	men women
	stupid b*tch	rohingya refugees
	illegal aliens	men like
	illegal immigrants	men men
	illegal alien	immigrant parents
3-gram	senkamalaharris hysterical woman	migrants https t
	build wall buildthatwall	refugees https t
	need wall buildthatwall	immigration https t
	speech time https	says https t
	free speech time	life https t
	buildthewall lockthemup enddaca	children https t
	trump maga rednationrising	woman accused nelly
	realdonaldtrump buildthewall lockthemup	accused nelly rape
	b*tch https t	today https t
	potus realdonaldtrump buildthewall	unitednations https t

TRAC(Facebook)

	NAG	CAG	OAG
Unigram	invest<br>good<br>buy<br>hi<br>cnbc<br>tata<br>nifty<br>proud<br>market<br>anuj	black<br>bike<br>bjp<br>burnol<br>mother<br>cash<br>old<br>modiji<br>men<br>reservation	islam<br>worst<br>fool<br>terrorist<br>hell<br>idiots<br>bloody<br>stupid<br>idiot<br>shame
2-gram	hi sonia<br>time buy<br>news jansatta<br>royal enfield<br>ratan tata<br>short term<br>mukesh ambani<br>hi anuj<br>bank nifty<br>long term	poor people<br>sonu right<br>ha ha<br>common people<br>old man<br>common man<br>indian express<br>political parties<br>modi ji<br>black money	dont forget<br>shame u<br>cheap publicity<br>u people<br>like u<br>gone mad<br>wrong decision<br>indian express<br>agent bjp<br>sonu nigam
3-gram	reason hcltech fall<br>advice bank nifty<br>bank nifty npa<br>hi anuj sonia<br>soul rest peace<br>real surgical strike<br>anuj sonia view<br>good time buy<br>long term view<br>cnbc tv 18	akhlaq killer draped<br>killer draped tricolor<br>black money holders<br>indian express mind<br>banned banned religions<br>good sonu nigam<br>common people suffering<br>surgical strike lol<br>owaisi mamta begum<br>trained kejriwal d	seriously need education<br>u seriously need<br>hate sonu nigam<br>old man useless<br>shame indian express<br>powerful man world<br>man world dont<br>9th powerful man<br>world dont forget<br>vote bank politics

HatebaseTwitter

	Hate	Offensive	Neither
Unigram	racist<br>queer<br>spic<br>f*g<br>f*gs<br>white<br>n*ggers<br>f*ggots<br>n*gger<br>f*ggot	c*nts<br>n*ccas<br>n*ggah<br>c*nt<br>sh*t<br>h*e<br>h*es<br>p*ssy<br>b*tches<br>b*tch	mock<br>oreo<br>colored<br>brownies<br>birds<br>trash<br>bird<br>yellow<br>charlie<br>yankees
2-gram	ugly d*ke<br>black people<br>you're f*cking<br>biggest f*ggot<br>stupid n*gger<br>n*gger music<br>f*ggot ass<br>you're f*ggot<br>f*cking f*ggot<br>white trash	b*tch i'm<br>ass b*tch<br>p*ssy http<br>yo b*tch<br>b*tch ass<br>bad b*tches<br>h*es http<br>h*e http<br>bad b*tch<br>like b*tch	derek jeter<br>rt yankees<br>like trash<br>charlie sheen<br>planet apes<br>trash talk<br>charlie crist<br>charlie brown<br>early bird<br>flappy bird
3-gram	vanessa f*ckin f*ggot<br>vinniepolitan kill coons<br>f*ggot rant night<br>amarierubio ch*nk eyed<br>n*ggah know wassup<br>runuldorants f*ggot sack<br>n*ggas retarded lmfaoo<br>happy birthday f*ggot<br>creepy ass cracker<br>south white trash	b*tch ass n*gga<br>don't love h*es<br>f*ck right p*ssy<br>like http t<br>b*tches http t<br>p*ssy http t<br>h*es http t<br>h*e http t<br>h*es ain't loyal<br>b*tch http t	yellow http t<br>brownies http t<br>new york yankees<br>early bird catches<br>bird catches worm<br>look like trash<br>bird http t<br>early bird gets<br>bird gets worm<br>trash http t
