# Supplementary figures and images for: Hate speech detection: Challenges and solutions
Source: PLoS One. 2019 Aug 20;14(8):e0221152. doi: 10.1371/journal.pone.0221152 (PMC6701757; doi:10.1371/journal.pone.0221152)

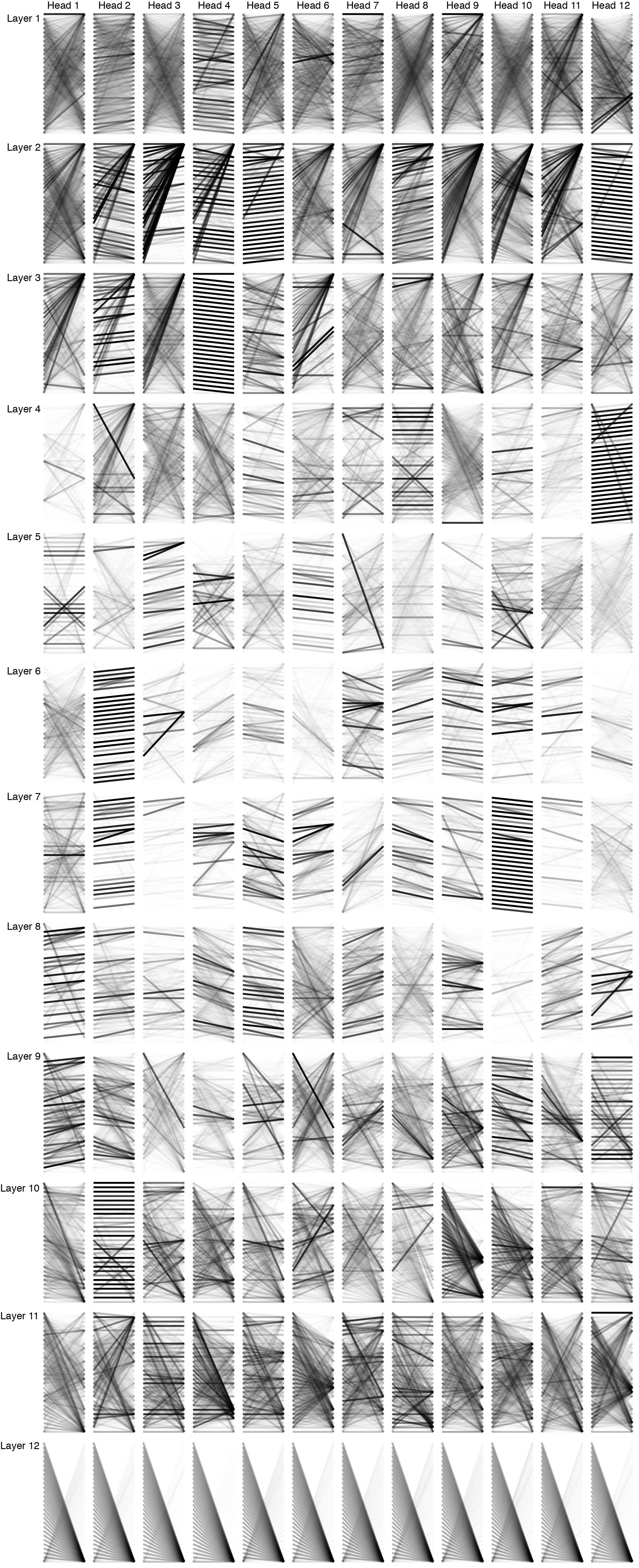

Supplement: S1 Fig — All layers and attention heads for the sentence “I don’t think anyone is insinuating that we are equal to non whites, or that we would ignore white nations.” are included. Darker lines indicate stronger attention between terms. The first token is the special classification token. (PNG) [file pone.0221152.s004.png]
